# New frontiers in the treatment of systemic juvenile idiopathic arthritis

**DOI:** 10.12688/f1000research.11327.1

**Published:** 2017-06-22

**Authors:** Susan Canny, Elizabeth Mellins

**Affiliations:** 1 Department of Pediatrics, Stanford University School of Medicine, Palo Alto, CA, USA; 2 Department of Pediatrics, Program in Immunology, Stanford University, Stanford, CA, USA

**Keywords:** systemic juvenile idiopathic arthritis, sJIA, macrophage activation syndrome, MAS, sJIA treatment

## Abstract

Systemic juvenile idiopathic arthritis (sJIA) and its most significant complication, macrophage activation syndrome (MAS), have traditionally been treated with steroids and non-steroidal anti-inflammatory medications. However, the introduction of biologic medications that inhibit specific cytokines, such interleukins 1 and 6, has changed the treatment paradigm for sJIA patients. In this review, we discuss the therapies currently used in the treatment of sJIA as well as novel targets and approaches under consideration, including mesenchymal stromal cell therapy and JAK inhibitors. We also discuss targeting cytokines that have been implicated in MAS, such as interferon gamma and interleukin 18.

## Introduction

Systemic juvenile idiopathic arthritis (sJIA) is classified as a subtype of JIA, although it is increasingly recognized as a distinct disease ^1– 4^. The ILAR classification criteria defines sJIA as arthritis in one or more joints, accompanied or preceded by systemic symptoms including quotidian fever of at least 2 weeks’ duration, an erythematous rash, lymphadenopathy, hepatomegaly, and splenomegaly and/or serositis but not associated with another diagnosis such as psoriasis, human leukocyte antigen (HLA)-B27 arthritis, inflammatory bowel disease, ankylosing spondylitis, or the presence of immunoglobulin (Ig) M rheumatoid factor ^5^. Estimates for the prevalence of JIA range from 16 to 400 cases per 100,000 children ^6^, with sJIA accounting for 4–17% of all JIA cases ^6^.

A proportion of children with sJIA will develop macrophage activation syndrome (MAS), with 10% developing an overt and potentially fatal clinical disease and 30–50% having occult MAS ^7– 10^. MAS is a form of secondary hemophagocytic lymphohistiocytosis (HLH) and accounts for the majority of the mortality associated with sJIA ^1, 9^. In 2016, new classification criteria for MAS in sJIA were defined, based on expert consensus and patient data, to classify patients for research studies. To be classified as having MAS, a patient must be febrile with a known or suspected diagnosis of sJIA and have a ferritin level greater than 684 ng/mL in addition to two of the following: platelet count ≤181 × 10 ^9^/L, aspartate aminotransferase (AST) >48 units/L, triglycerides >156 mg/dL, and/or fibrinogen ≤360 mg/dL ^11, 12^.

sJIA can proceed with a monophasic, polycyclic (periods of flare separated by periods of remission), or persistent course of disease ^1, 13^. When remission is defined as inactive disease off medications for at least 3 months, most patients will have either a monophasic or a persistent disease course. In one prospective cohort study, 42.2% of patients had a monophasic course, 6.7% of patients had a polycyclic course, and 51.1% of patients had persistent disease ^13^. Features associated with persistent disease include polyarticular arthritis early in disease and persistence of disease activity (specifically arthritis, elevated erythrocyte sedimentation rate [ESR], and use of corticosteroids) at 3 and 6 months ^13^. Persistent disease can be further subdivided into either predominately systemic or arthritic disease. Children with sJIA who develop persistent arthritis only (often referred to as systemic onset, polyarticular course) may represent a distinct subtype of sJIA and may benefit from distinct treatment approaches ^14^. In a recent cross-sectional analysis of North American sJIA patients, this subtype typically had more functional disability, despite a shorter time to diagnosis, and had longer disease duration, consistent with the possibility that, in some patients, sJIA evolves into this phenotype over time ^14^.

Recent data from a genome-wide association study of sJIA suggest that sJIA has a genetic architecture that is distinct from other forms of JIA ^2^. Whereas other subtypes of JIA have features of classic autoimmune diseases, sJIA may be better described as sharing features of both autoinflammatory and autoimmune diseases ^1, 15– 17^. Autoinflammatory diseases are mediated by cells of the innate immune system and inflammatory cytokines, such as interleukin (IL)-1 and IL-6, in contrast to the classical autoimmune diseases, which are mediated by cells of the adaptive immune system and are frequently found to be associated with specific HLA alleles ^15, 18^. Several studies suggest a role for natural killer (NK) cells, part of the innate immune system, in sJIA, particularly during MAS ^19– 24^. In the most recent study, analysis of RNA sequencing data from sJIA NK cells revealed an enrichment of inflammatory pathways with downregulation of IL-10 receptor A and granzyme K ^23^.

A recent study by Ombrello *et al*. described an HLA gene association (HLA-DRB1*11) with sJIA ^16^. Class II major histocompatibility complex (MHC) molecules are expressed on professional antigen-presenting cells and interact with CD4 ^+^ T cells via the αβ T cell receptor, but these molecules may also play a role in the regulation of innate responses ^25, 26^. As the authors note, the HLA association may reflect a contribution to sJIA pathogenesis via CD4 ^+^ T cells and/or via “dysregulation of innate immunity” ^16^. Nigrovic has proposed a biphasic model of sJIA in which innate immune factors dominate the initial disease presentation, whereas adaptive immune components, such as a skewing of the CD4 ^+^ T cell population to favor Th17 over regulatory T cell development, contribute in those patients in whom chronic arthritis develops ^17^. The possibility that acute systemic disease is dominated by innate factors and chronic arthritis is dominated by adaptive immune factors suggests that different treatment approaches may be warranted in different phases of disease ^17^.

## Treatment

Traditionally, sJIA has been treated with a combination of non-steroidal anti-inflammatory medications, such as indomethacin, and steroids. In the pre-biologic era, studies of long-term outcome reported 40–50% of sJIA patients followed for at least 10 years still had active disease ^27^. Data on the efficacy of high-dose intravenous Ig (IVIG) are conflicting ^28, 29^. In practice, IVIG was often tried because of lack of steroid response or steroid toxicity, but, currently, IVIG is used less often ^30^. Methotrexate, a first-line treatment in other subtypes of JIA, has also been used for sJIA. Indeed, current recommendations from the American College of Rheumatology (ACR) include methotrexate as an option for sJIA patients with persistent arthritis ^30^. The only randomized, placebo-controlled trial of (low-dose, oral) methotrexate evaluated a group of children with persistent arthritis for >1 year, many of whom also had signs of active systemic disease. The trial did not demonstrate a significant difference between methotrexate and placebo in joint scores or systemic features, suggesting that its utility in sJIA may be limited ^31^.

The advent of biologic medications has provided new avenues for the treatment of sJIA and options for steroid-sparing therapies ^3^. Several studies have demonstrated the efficacy of inhibitors of the inflammatory cytokines IL-1 and IL-6 ^3, 32– 37^. Trials of canakinumab, a monoclonal antibody to IL-1β, and tocilizumab, a monoclonal antibody to the IL-6 receptor, showed significant clinical improvement in patients treated with these medications ^32, 33^. A total of 84% of patients treated with canakinumab demonstrated clinical response compared to 10% in the placebo group ^33^. Similarly, 85% of patients treated with tocilizumab had clinical improvement compared to 24% in the placebo group ^32^. Several recent reviews nicely present further details of therapies that inhibit IL-1 and IL-6 ^38, 39^. Increased susceptibility to infections is observed in patients treated with these medications ^32, 33, 40^. The most common reported infections include nasopharyngitis, upper respiratory tract infections, bronchitis, and gastroenteritis, although serious bacterial infections including pneumonia and sepsis in addition to rare cases of herpesvirus infections including varicella, herpes zoster, cytomegalovirus, and Epstein-Barr virus have been reported ^32, 33, 37, 40– 43^.

The facts that some patients respond to biologics and others do not and that the latter group may be enriched for those with long-standing disease have given rise to a hypothesis that there is a “window of opportunity” for treatment, i.e. if patients are treated early with agents that block IL-1 or IL-6, this may modulate the course of their disease ^17, 37, 44, 45^. In an uncontrolled trial of anakinra (IL-1 receptor antagonist) as first-line therapy in 20 steroid-naïve patients, >80% developed inactive disease, either on or off medication, that persisted over a mean follow-up of >2 years ^44^. However, the fraction of these patients with monocyclic disease that would have remitted without biologic therapy is not known. Currently, it remains uncertain whether there is truly a window of opportunity or a subset of patients in whom such treatments are effective.

Although many patients respond to IL-1 and IL-6 inhibition, a subset of patients continues to have refractory sJIA. Nearly one-third of patients develop persistent polyarthritis after resolution of the systemic symptoms ^14^. For these patients, medications including abatacept, a CTLA-4 fusion protein that blocks T cell co-stimulation via CD80/CD86, have demonstrated some efficacy ^46, 47^. Several sJIA patients with refractory disease, including some with persistent arthritis, were treated with abatacept in combination with anakinra with clinical improvement and ability to reduce doses of anakinra and steroids ^47^. The fact that abatacept, which blocks T cell activation, is effective in some refractory sJIA cases suggests new therapeutic targets for these patients and a possible role for T cells in refractory disease.

There is no current consensus on the optimal way to treat recurrent MAS in sJIA. A range of approaches is used from a combination of steroids, IL-1 inhibitors, and cyclosporine to etoposide with steroids (based on HLH treatment protocols) to stem cell transplantation. Treatment protocols for HLH developed by the Histiocyte Society include etoposide and steroids with or without cyclosporine and intrathecal methotrexate and steroids followed by bone marrow transplantation for patients with familial HLH or refractory secondary HLH ^48– 51^. In patients with sJIA, the emphasis typically has been on controlling the underlying disease ^48^. However, it is worth noting that MAS, triggered by infections, occurs even when patients’ underlying sJIA is controlled ^42^. As discussed below, new approaches may hold promise for the treatment of refractory MAS, given their efficacy in animal models of HLH.

Hematopoietic stem cell transplantation (HSCT) has been used to treat severe refractory sJIA ^52– 54^. Following several deaths in transplant patients due to virally induced MAS, investigators aimed to achieve improved control of disease prior to transplantation; the conditioning protocol was amended to mitigate T cell depletion, and patients were placed on antiviral prophylaxis after transplantation ^52^. Data suggest that HSCT allows for a resetting of the immune regulatory network with a recovery of regulatory T cells ^55^.

## On the horizon

Given the risks associated with HSCT and the advent of biologic medications, the use of allogeneic stem cell transplants in sJIA has declined ^56^. Investigators are now exploring the possibility of multipotent mesenchymal stromal cell (MSC) therapy as an alternative for the treatment of autoimmune diseases refractory to other treatments. Studies have shown that MSCs have immunomodulatory properties and are well tolerated ^56– 59^. In 10 patients with JIA treated with MSCs, markers of inflammation (ESR and C-reactive protein [CRP]) were significantly decreased and regulatory T cells were significantly increased ^60^. Additional larger studies are warranted.

Evidence suggests that the cytokine interferon gamma (IFNγ) plays an important role specifically in MAS ^38, 61, 62^. Levels of IFNγ and IFNγ-induced genes are not elevated in patients with sJIA without signs of MAS ^63– 65^. In contrast, in patients with sJIA complicated by MAS, IFNγ levels and IFNγ-induced gene expression increase ^61, 62^. A phase II/III trial is currently underway to assess the efficacy of an anti-IFNγ monoclonal antibody in primary HLH. Based on the evidence for a role for IFNγ in MAS, targeting IFNγ in secondary HLH may also prove fruitful.

The cytokine IL-18 is another potential target in sJIA, as studies have shown that patients with sJIA have high levels of IL-18 and substantially increased levels may be associated with risk of MAS ^66– 70^. The IL-18 binding protein (IL-18BP) regulates IL-18 action; it binds IL-18 with high affinity and inhibits its activity ^71^. Administration of synthetic IL-18BP in a mouse model of HLH (perforin-1-knockout mice infected with murine cytomegalovirus) diminished liver and spleen damage and reduced levels of the inflammatory cytokines IFNγ and TNFα, although it did not alter survival ^72^. Notably, elevated levels of IL-18 persist for at least a few months in clinical remission of sJIA ^66, 67^, suggesting that further research is needed to clarify the role of IL-18 in disease pathogenesis and control of disease. A recent case report has demonstrated the potential utility of recombinant IL-18BP in a patient with an MAS-like syndrome due to mutation of NLRC4, a protein that triggers activation of the inflammasome ^73^. Whether IL-18BP will be effective clinically in sJIA or MAS remains to be seen and, if so, whether the timing of administration determines clinical response.

Another possible cytokine target is IL-17. As noted above, Th17 cells have been proposed to play a pathogenic role in the chronic arthritic phase of sJIA ^17^. IL-17-producing T cells (CD4 ^+^ and CD4 ^–^) have been found at higher proportions in the peripheral blood of sJIA patients compared to healthy, age-matched controls ^74^. Biologic agents modulating the action of IL-17 are in various stages of investigation in the treatment of psoriasis, psoriatic arthritis, and rheumatoid arthritis ^75^ and may be worth considering in the treatment of sJIA patients with chronic arthritis. ****

Cytokines signal through their cell surface receptors to induce changes in gene transcription. Signaling molecules include the enzymes in the Janus kinase (JAK) family. New small molecule inhibitors of JAKs are under development. Tofacitinib, an inhibitor of JAK1 and JAK3, has been found to be effective in adults with rheumatoid arthritis ^76^. Varicella zoster virus reactivation and cytomegalovirus infection have been reported in patients on tofacitinib, although no cases of Epstein-Barr virus, a common MAS trigger, have been reported ^77, 78^. Given the association of viral triggers with MAS, JAK inhibitors, at least tofacitinib, may be less attractive as therapeutic options in patients at risk of developing MAS ^20, 42, 79, 80^. In contrast, ruxolitinib, an inhibitor of JAK1 and JAK2, has been tested in mouse models of HLH and found to promote survival and reduce levels of pro-inflammatory cytokines IL-6 and TNFα ^81, 82^, suggesting that JAK1/2 may be a therapeutic target in patients with HLH. Whether these small molecule JAK inhibitors will be effective in sJIA or MAS in children requires further study.

Given the small numbers of patients with sJIA, collaborative research and research networks, such as the Childhood Arthritis and Rheumatology Research Alliance (CARRA), the Paediatric Rheumatology International Trials Organisation (PRINTO), and the Pediatric Rheumatology Collaborative Study Group (PRCSG), will likely play an important role in developing the next generation of sJIA treatments. CARRA has developed consensus treatment plans for sJIA to be used in comparative efficacy trials of different treatments ^83, 84^. The ACR has recently developed comprehensive guidelines for the treatment of sJIA based on disease activity and disease phenotype ^30^. Understanding the pathogenesis of sJIA and its complications, including MAS and refractory arthritis, will be important in defining new therapeutic targets and ensuring that clinical treatments optimize therapeutic outcomes while minimizing treatment toxicities.
